# Determinants of infant mortality in Oromia region, Ethiopia

**DOI:** 10.1097/MS9.0000000000000842

**Published:** 2023-05-12

**Authors:** Tariku Irana, Gizachew Gobebo Mekebo, Gezahagn Diriba, Assefa Legesse Sisay, Birhanu Woldeyohannes, Zemene Yohannes

**Affiliations:** a College of Natural and Computational Science, Ambo University, Ambo; b Institute of Health Science, Jimma University, Jimma; c Technical and Financial Team Leader, Addis Ababa Transport Bureau; d Assistant Researcher in Industrial Development Policy Studies Centre, Ethiopian Policy Studies Institute, Addis Ababa, Ethiopia

**Keywords:** 2019 EMDHS, determinants, ethiopia, infant mortality, oromia region

## Abstract

Infant mortality is one of the commonest health-related indicators used to assess the health status of the community. Children born in Sub-Saharan Africa are at highest risk of infancy death in the world. Ethiopia, in Sub-Saharan Africa, is a country with high infant mortality. Oromia region is among the regions with high infant mortality rate in Ethiopia. Therefore, this study aimed to identify determinants of infant mortality in Oromia region, Ethiopia.

**Methods:**

The source of data for this study was 2019 Ethiopian Mini Demographic and Health Survey. Multivariable logistic regression model was employed to identify the determinants the infant mortality. An adjusted odds ratio (OR) with a 95% CI was used examine the determinants of infant mortality.

**Results:**

A total of 719 live births born 5 years preceding the survey were included. The infant mortality rate in the study area was 54 deaths per 1000 live births. The risk of dying in infancy was lower for females [adjusted OR (AOR): 0.518, 95% CI: 0.284, 0.945], health deliveries (AOR: 0.429, 95% CI: 0.235, 0.783), infants born to mothers attended ANC during pregnancy (AOR: 0.603, 95% CI: 0.489, 0.744), infants from families with wealth indices of medium (AOR: 0.715, 95% CI: 0.580, 0.882) and rich (AOR: 0.638, 95% CI: 0.425, 0.958) compared with the respective reference categories while it was higher for infants of multiple births (AOR: 2.241, 95% CI: 1.768, 2.841) compared with singletons.

**Conclusions:**

Infant mortality rate in the study area, Oromia region, is higher than the national figure. The study found that sex of child, birth type, antenatal care (ANC), place of delivery and wealth index of household were significant determinants of infant mortality. Therefore, concerned bodies should make awareness creation to mothers regarding ANC and encourage them to have ANC follow-up during pregnancy and deliver at health institution to improve the infant survival in the region.

## Introduction

Highlights

- This study aimed to identify determinants infant mortality in Oroimia Region, Ethiopia.
- Analysis was based on 2019 Ethiopian Mini Demographic and health Survey data.
- Multivariable logistic model was employed to identify the determinants infant mortality.
- The study found that sex of child, birth type, antenatal care (ANC) visit, place of delivery and wealth index of household were significant determinants of infant mortality.
- Concerned bodies should make awareness creation to mothers regarding ANC and encourage them to have ANC follow-up during pregnancy and deliver at health institution to improve the infant survival in the region.

Infant mortality, the death of a child during the first year of life^1^, is one of the commonest health-related indicators used to assess the health status of the community^2,3^.

There has been global remarkable decline in infant mortality rate from 65 deaths per 1000 live births in 1990 to 28 deaths per 1000 live births in 2021^4^. Despite this remarkable decline worldwide, Sub-Saharan Africa and Southern Asia remain the regions with the burden of child deaths falls hardest. Children born in sub-Saharan Africa continue to suffer the world’s highest mortality rates^5^.

In 2021, Sub-Saharan Africa with 50 deaths per 1000 live births is a region with highest infant mortality rate compared to other Sustainable Development Goal regions^5^. The infant mortality rate in Ethiopia was 34 deaths per 1000 deaths in 2021^4^.

The prevalence of infant mortality is higher in those regions where infants are particularly vulnerable to disease like malaria, diarrhoea, asphyxia, malnutrition and pneumonia^4^. The causes of infant mortality are highly associated with social wellbeing, quality of the environment, economic development, general living conditions and preventable bio-demographic factors^6–8^.

According to the reports of previous researchers, infant mortality is determined by factors such as maternal education level^8–13^, maternal age^14–17^, maternal age at first birth^18,19^, maternal anaemia^20,21^, diarrhoea^22,23^, sex of child^7,10,11,13,15,24^, marital status, desire of pregnancy, residence, region, maternal HIV^13,15,25–34^, birth type, birth order, preceding birth interval, birth size^7,10,11,14,21,24,31,32^, breastfeeding status, breastfeeding initiation^6,15,24^, gestational age, vaccination status and antenatal care (ANC) visits^4,12,27,35^, place of delivery^7,11,24,35^, use of family planning^12^, employment status, wealth status^5,13,27,31,36,37^, family size^13,24^, parity^21^, source of water and household electricity^7,12,38,39^.

The most recent studies in Ethiopia and other African countries also show that nutritional status of child, birth asphyxia, distance to health facility, age of infant, maternal medical complications during pregnancy, prematurity, maternal death at birth, maternal education, maternal age, birth type and sex of child^40–45^ were determinants of infant mortality.

According to the 2019 Ethiopian Mini Demographic and Health Survey (2019 EMDHS) report, infant mortality rate for the 5 years before the survey is 47 deaths per 1000 live births, which is equivalent to 1 in every 21 die before their first birthday. This result shows that infant mortality declined from 77 deaths per 1000 live births in 2005 to 47 deaths per 1000 live births in 2019, a 39% reduction over the past 14 years^40^. Even more efforts are needed to be made in Ethiopia to reduce infant mortality rate to the level targeted by sustainable development goal to be attained by 2030. The 2019 EMDHS report also shows that infant mortality rate in Oromia region is 62 deaths per 1000 live births, which is higher than the national figure, 47 deaths per 1000 live births^46^. Various studies on infant mortality have been conducted in Ethiopia; however, to the best of our knowledge, no study has been done at the regional level in the Oromia region using the national representative data. Therefore, this study aimed to identify the determinants of infant mortality in Oromia region, Ethiopia using 2019 EMDHS data.

## Methods

### Data source, study design and sample size

Data used for this study were obtained from 2019 EMDHS data, which is a national representative cross-sectional survey data collected from 21 March to 28 June 2019. The 2019 EMDHS is the second EMDHS to be implemented in Ethiopia^46^. The study specifically used the data from 2019 EMDHS kids’ data belonging to Oromia region. A sample of 719 children was included in the study.

### Study variables

Response variable was death of live born before first birthday, which was coded as 1 if the live born died before first birthday and 0 otherwise, and independent variables were sex of child, birth type, birth order, ANC visits, place of residence, maternal age, maternal age at first birth, maternal education level, mother’s marital status, religion, place of delivery, family size, parity, sex of household head and wealth status of household.

### Statistical analysis

Data were analysed by SPSS version 26. The data were analysed using both descriptive and inferential statistics. Frequencies and percentages were used to summarise background characteristics of the participants. Both bivariate logistic regression analysis and Multivariable logistic regression analysis were done. Bivariate analysis was conducted to select candidate variables for multivariable logistic regression model. The multivariable logistic regression model was used to identify the determinants of infant mortality. The significance was considered at *P* value less than 0.05. Goodness of fit of the model was checked using Hosmer and Lemeshow test and the model with significance value of 0.595 found to be good fit.

## Results

### Characteristics of study participants and bivariate analysis of infant mortality

Of the total of 719 live births included in this study, 39 (5.4%) were died before celebrating their first birth days indicating that infant mortality rate in the study area was 54 deaths per 1000 live births. About 51.2% of the children were males. Nearly nine-tenth (87.9%) were born in rural areas. Majority (98.1%) of the children were singletons. About two-fifth (40.6%) of them had birth order of second to fourth. Nearly three-fifth (58.6%) were born at home. About four-fifth (79.8%) of them were born to mothers who had ANC visits. Regarding mother’s age, nearly four-fifth (78.9%) of mothers aged less than 25 years. More than half (53.8%) of them aged less than 20 years at their first births. Majority (95.1%) of them were in union at a time of survey. Slightly more than half (52.3%) of them had not attended education at all and only 7.2% of them attended secondary and higher education. Nearly half (49.1%) of them were from households with wealth indices of poor (Table 1).

**Table 1:** Characteristics of infants and mothers, and bivariate analysis result of infant mortality in Oromia region, Ethiopia, 2019 EMDHS

			Status of infant		
Variables	Categories	Count, *n*, (%)	Died before 1^st^ birthday, *n*, (%)	Not died before 1^st^ birthday, *n*, (%)	*P*
Sex of infant	Male	368 (51.2)	25 (6.8)	343 (93.2)	
	Female	351 (48.8)	14 (4.0)	337 (96.0)	0.018^*^
Birth type	Single	705 (98.1)	37 (5.2)	668 (94.8)	
	Multiple	14 (1.9)	2 (14.3)	12 (85.7)	0.006^*^
Birth order	1^st^	147 (20.4)	9 (6.1)	138 (93.9)	
	2^nd^–4^th^	292 (40.6)	15 (5.1)	277 (94.9)	0.669
	5^th^ and above	280 (38.9)	15 (5.4)	265 (94.6)	0.744
Place of delivery	Home	421 (58.6)	20 (6.4)	401 (95.2)	
	Health institution	298 (41.4)	19 (4.8)	279 (93.6)	0.025^*^
ANC visits	No	145 (20.2)	11 (7.6)	134 (92.4)	
	Yes	574 (79.8)	28 (4.9)	546 (95.1)	0.002^*^
Place of residence	Urban	87 (12.1)	2 (2.3)	85 (97.7)	
	Rural	632 (87.9)	37 (5.9)	595 (94.1)	0.040^*^
Maternal age	Less than 25	567 (78.9)	33 (5.8)	534 (94.2)	
	25–34	29 (4.0)	1 (3.4)	28 (96.6)	0.596
	35+	123 (17.1)	5 (4.1)	118 (95.9)	0.442
Age of mother at first birth					
	Less than 20	387 (53.8)	22 (5.7)	365 (94.3)	
	20–24	180 (25.0)	11 (6.1)	169 (93.9)	0.840
	25+	152 (21.2)	6 (3.9)	146 (96.1)	0.416
Mother’s marital status	Never in union	2 (0.3)	1 (50.0)	1 (50.0)	
	Currently in union	684 (95.1)	37 (5.4)	647 (94.6)	0.035^*^
	Widowed/separated	33 (4.6)	1 (3.0)	32 (97.0)	0.022^*^
Religion	Orthodox	85 (11.8)	5 (5.9)	80 (94.1)	
	Protestant	219 (30.5)	7 (3.2)	212 (96.8)	0.288
	Others	415 (57.7)	27 (6.5)	388 (93.5)	0.831
Maternal education level	No education	376 (52.3)	18 (4.8)	358 (95.2)	
	Primary	291 (40.5)	16 (6.5)	272 (93.5)	0.332
	Secondary and higher	52 (7.2)	2 (3.8)	50 (96.2)	0.764
Family size	Less than 5	203 (28.2)	18 (8.9)	185 (91.1)	
	5+	519 (71.8)	21 (4.1)	495 (95.9)	0.471
Parity	1–3	318 (44.2)	15 (4.7)	303 (95.3)	
	4–5	167 (23.2)	11 (6.6)	156 (93.4)	0.387
	6+	234 (32.5)	13 (5.6)	221 (94.40	0.658
Sex of household head	Male	661 (91.9)	34 (5.1)	627 (94.9)	
	Female	58 (8.1)	5 (8.6)	53 (91.4)	0.262
Wealth status of household	Poor	353 (49.1)	18 (5.1)	335 (94.6)	
	Medium	165 (22.9)	11 (6.7)	154 (93.3)	0.013^*^
	Rich	201 (28.0)	10 (5.0)	191 (95.0)	0.008^*^

1: Reference group,

ANC, antenatal care; EMDHS, Ethiopian Mini Demographic and Heath survey.

* Significant.

Table 1 also shows that percentage of infant mortality was higher among males (6.8%). There was also higher percentage of infant mortality among multiple births (14.3%). The percentage of infant mortality was higher among infants born from mothers residing in rural areas (5.9%). Higher percentage of infant mortality (6.4%) was observed among infants born at home. Higher percentage of infant mortality was also observed among infants born to mothers who did not attend ANC (7.6%).

### Factors associated with infant mortality in Oromia region, Ethiopia

The result multivariable logistic regression analysis shows that sex of infant, birth type, ANC visit, place of delivery and wealth status of household significantly determinant infant mortality at 5% level of significance. The odds of infant mortality among females was 0.518 [adjusted odds ratio (AOR): 0.518, 95% CI: 0.284, 0.945] times lower than males. The odds of infant mortality among infants of multiple births was 2.241 (AOR: 2.241, 95% CI: 1.768, 2.841) times higher than infants of single births. Infants born in health institutions were (AOR: 0.429, 95% CI: 0.235, 0.783) less likely to die than infants born at home. Infants born to mothers who visited ANC were (AOR: 0.603, 95% CI: 0.489, 0.744) times less likely to die than infants born mothers who did not attend ANC. Infants from families with wealth indices of medium and rich were (AOR: 0.715, 95% CI: 0.580, 0.882) and (AOR: 0.638, 95% CI: 0.425, 0.958) times, respectively, less likely to die than infants from poor families (Table 2).

**Table 2:** Factors associated with infant mortality in Oromia region, Ethiopia, 2019 EMDHS.

Variables	Categories	AOR (95% CI)	*P* (AOR)
Sex of infant	Male	1	
	Female	0.518 (0.284, 0.945)	0.037^*^
Birth type	Single	1	
	Multiple	2.241 (1.768, 2.841)	0.020^*^
Place of delivery	Home	1	
	Health institution	0.429 (0.235, 0.783)	0.042^*^
ANC visits	No	1	
	Yes	0.603 (0.489, 0.744)	0.005^*^
Place of residence	Urban	1	
	Rural	2.699 (0.697, 6.124)	0.158
Mother’s marital status	Never in union	1	
	Currently in union	0.480 (0.219, 1.053)	0.067
	Widowed/separated	0.549 (0.274, 1.013)	0.091
Wealth status of household	Poor	1	
	Medium	0.715 (0.580, 0.882)	0.040^*^
	Rich	0.638 (0.425, 0.958)	0.027^*^

1, Reference group.

ANC, antenatal care; AOR, adjusted odds ratio; EMDHS, Ethiopian Mini Demographic and Heath survey.

* Significant.

## Discussion

This study was aimed to identify determinants of infant mortality in Oromia region, Ethiopia. A total of 719 live born, of which 39 (5.4%) were died before celebrating first birthday, were included in the study. The multivariable logistic regression analysis revealed that sex of infant significantly determines infant mortality. Female babies were at lower risk of infancy mortality than males. This could be due to that girls are biologically stronger and less susceptible to diseases and death. This agrees with the results of studies^7,13,21,24,32,45,47^.

In this study, place of delivery was also found to be significant determinant of infant mortality. Infants born in health institutions were less likely to die than infants born at home. This is consistent with studies^7,12,24,36,38,39^. This might be due to the immediate access to care among babies born at health institutions by skilled birth attendants.

This study also found that infants born to mothers who had ANC visit were less likely to die than infants born to mothers who had no ANC visit. This might be because of that mother who had ANC visit may be aware of any danger signs of pregnancy and underlying medical conditions and may take treatment for the problems that can improve the quality of infant life. This result agrees with studies^21,48^. In addition, the study found that infant mortality decreases with increased wealth status of household. This result is consistent with studies^13,31,49,50^.

In the study, it was also found that multiple births infants were at higher risk of death than single infants before celebrating first birthday. This result is consistent with results of the studies^7,10,21,24,32,36,45^. The possible reason could be that twins are more likely to be born prematurely, and lower birth weight than single birth infants^51^.

### Strength and limitation of the study

The main strength of this study was that it was based on the national representative survey data. One of the limitations of the study was that it did not include variables such as size of child at birth, mothers working status, educational status of father, preceding birth interval, and breastfeeding status, that might determine infant mortality due to high missing values and unavailability in the dataset. The other limitation was that the sample size of 39 deaths was very small that might affect conclusion.

## Conclusion

The overall infant mortality rate in this study was 54 deaths per 1000 live births. Infant mortality rate among male babies was 68 deaths per 1000 live births. It was 59 deaths per 1000 live births among babies born in rural areas. Infant mortality rate among multiple births was 143 deaths per 1000 live births. Infant mortality rate among home delivered babies was 64 deaths per 1000 live births and it was 76 deaths per 1000 live births among babies born from mothers who did not attend ANC during their pregnancies. It was found that sex of child, birth type, ANC visit, place of delivery and wealth index of household were significant determinants of infant mortality in Oromia region, Ethiopia. Male babies were at higher risk of dying before first birthday compared to female babies. Babies of multiple births were at higher risk of dying before first birthday compared to singletons. Babies born at health institutions were at lower risk of dying before first birthday compared with those born at home. The risk of infancy death was lower among babies born to mothers who had ANC visits than those born to mothers who did not have ANC visits during their pregnancies. Babies born from poor families were at higher risk of dying before first birthday compared with those born from families with wealth indices of medium and rich. Therefore, concerned bodies should make awareness creation to mothers regarding ANC, and encourage them to have ANC follow-up during pregnancy and deliver at health institution to improve the infant survival in the region. In addition, further studies need to be conducted considering those factors which were not included in this study because of unavailability/high missing values in the dataset we used.

## Ethical approval and consent to participate

The data are secondary data and publicly available and has no personal identifiers.

## Consent

Not applicable.

## Sources of funding

This study was not funded.

## Conflicts of interest disclosure

Authors declare that they do not have conflict of interest.

## Author contribution

T.I.: conceptualization, data curation, formal analysis, investigation, methodology, software, validation, writing—original draft. G.G.M.: conceptualization, formal analysis, investigation, methodology, software, validation, writing—review and editing. G.D.: data curation, formal analysis, investigation, methodology, software, writing—review and editing. A.L.S., B.W. and Z.Y.: formal analysis, methodology, software, writing—review and editing.

## Research registration unique identifying number (UIN)

NA.

## Guarantor

Gizachew Gobebo Mekebo.

## Data availability

All data used for the analysis in this study are available upon reasonable request from corresponding author.

## Provenance and peer review

Not commissioned, externally peer-reviewed

## References

[1] National Commission to Prevent Infant Mortality (US). Death Before Life: the Tragedy of Infant Mortality: the Report of the National Commission to Prevent Infant Mortality. Commission 1988:7–39.

[2] Deogaonkar M . Socio-economic inequality and its effect on healthcare delivery in India: inequality and healthcare. Electron J Soc 2004;11:25–35.

[3] Reidpath DD Allotey P . Infant mortality rate as an indicator of population health. J Epidemiol Community Health 2003;57:344–346. 12700217 10.1136/jech.57.5.344 PMC1732453

[4] United Nations Inter-agency Group for Child Mortality Estimation (UN IGME). Levels & Trends in Child Mortality: Report 2022, Estimates developed by the United Nations Inter-agency Group for Child Mortality Estimation. United Nations Children’s Fund; 2023.

[5] United Nations Inter-agency Group for Child Mortality Estimation (UN IGME). ‘Levels & Trends in Child Mortality: Report 2021, Estimates developed by the United Nations Inter-agency Group for Child Mortality Estimation’. United Nations Children’s Fund; 2021.

[6] D Allotey P . Theory and methods infant mortality rate as an indicator of population health. J Epidemiol Community Health 2003;57:344–346. 12700217 10.1136/jech.57.5.344 PMC1732453

[7] Tiruneh SA Zeleke EG Animut Y . Time to death and its associated factors among infants in sub-Saharan Africa using the recent demographic and health surveys: shared frailty survival analysis. BMC Pediatr 2021;21:1–3. 34607560 10.1186/s12887-021-02895-7 PMC8489062

[8] Black RE Morris SS Bryce J . Where and why are 10 million children dying every year? Lancet 2003;361:2226–2234. 12842379 10.1016/S0140-6736(03)13779-8

[9] Moazzem Hossain SM El Nakib S Ibrahim S . Maternal and neonatal health in select districts of Iraq: findings from a recent household survey. J Pregnancy Child Health 2018;5:10–4172.

[10] Abate MG Angaw DA Shaweno T . Proximate determinants of infant mortality in Ethiopia, 2016 Ethiopian demographic and health surveys: results from a survival analysis. Arch Public Health 2020;78 1:1–0. 31993199 10.1186/s13690-019-0387-4 PMC6975059

[11] Regesa BH Senbeto T Gobebo G . Analysis of Determinants of Neonatal Mortality in Afar and Somalia Regions, Ethiopia. J Pharm Res Int [Internet] 2022;34(1B):63–71.

[12] Gupta N Hirschhorn LR Rwabukwisi FC . Causes of death and predictors of childhood mortality in Rwanda: a matched case-control study using verbal social autopsy. BMC Public Health 2018;18:1–9. 10.1186/s12889-018-6282-z PMC6296058 30558586

[13] Shibre G . Social inequality in infant mortality in Angola: evidence from a population based study. PLoS One 2020;15:e0241049. 33091077 10.1371/journal.pone.0241049 PMC7580929

[14] Kusneniwar GN Mishra AK Balasubramanian K . Determinants of infant mortality in a developing region in rural Andhra Pradesh. Natl J Integr Res Med 2013;4:20. 24817796 PMC4012071

[15] Handiso A Mekebo GG Galdassa A . Trends and determinants of road traffic accident human death in Kembata Tembaro zone, SNNPR, Ethiopia. Science Journal of Applied Mathematics and Statistics 2022;10: 85–89.

[16] Finlay JE Özaltin E Canning D . The association of maternal age with infant mortality, child anthropometric failure, diarrhoea and anaemia for first births: evidence from 55 low-and middle-income countries. BMJ Open 2011;1:e000226. 10.1136/bmjopen-2011-000226 PMC3191600 22021886

[17] Kim YN Choi DW Kim DS . Maternal age and risk of early neonatal mortality: a national cohort study. Sci Rep 2021;11:1–9. 33436971 10.1038/s41598-021-80968-4 PMC7804272

[18] Ahinkorah BO . Maternal age at first childbirth and under-five morbidity in sub-Saharan Africa: analysis of cross-sectional data of 32 countries. Arch Public Health 2021;79:1–0. 34425906 10.1186/s13690-021-00674-5 PMC8383451

[19] Neal S Channon AA Chintsanya J . The impact of young maternal age at birth on neonatal mortality: evidence from 45 low and middle income countries. PLoS One 2018;13:e0195731. 29791441 10.1371/journal.pone.0195731 PMC5965834

[20] Argawu AS Mekebo GG Bedane K . Prevalence and determinants of anaemia among reproductive-aged women in Ethiopia: a Nationally Representative Cross-sectional Study. J Pharm Res Int 2021;33(59B):687–698.

[21] Tesema GA Seretew WS Worku MG . Trends of infant mortality and its determinants in Ethiopia: mixed-effect binary logistic regression and multivariate decomposition analysis. BMC Pregnancy Childbirth 2021;21:1–6. 33952208 10.1186/s12884-021-03835-0 PMC8097868

[22] Kefale B Bedada D Negash Y . Determinants of diarrhea among children under age five using generalized linear model with Bayesian approach: the Case of Kuyu General Hospital, Oromia Region, Ethiopia. Clinics Mother Child Health S 2021;18:1–9.

[23] WHO: Children: improving survival and well-being. Geneva: WHO; 2020. https://www.who.int/news-room/fact-sheets/detail/children-reducing-mortality on 11 Feb 2021 .

[24] Gobebo G . Determinant factors of under-five mortality in Southern Nations, Nationalities and People’s region (SNNPR), Ethiopia. Ital J Pediatr 2021;47:1–9. 34717721 10.1186/s13052-021-01118-0 PMC8557013

[25] Wolde TS Mekebo GG . Unintended pregnancy and associated factors among pregnant women in rural Ethiopia. JPRI 2021;33(60B):2432–2440.

[26] Argawu AS Mekebo GG . Risk factors of under-five mortality in Ethiopia using count data regression models, 2021. Ann Med Surg 2022;82:104764. 10.1016/j.amsu.2022.104764 PMC9577841 36268401

[27] Alam M Islam M . Is there any association between undesired children and health status of under-five children? Analysis of a nationally representative sample from Bangladesh. BMC Pediatr 2022;22:1–4. 35879700 10.1186/s12887-022-03489-7 PMC9310505

[28] Arunda MO Choudhry V Ekman B . Under-five mortality and maternal HIV status in Tanzania: analysis of trends between 2003 and 2012 using AIDS Indicator Survey data. Global Health Action 2016;9:31676. 27329937 10.3402/gha.v9.31676 PMC4916291

[29] Gobebo G Siffir A Hailu B . Influencing factors of mortality among adult HIV patients under antiretroviral therapy: the Case of Hossana Queen Elleni Mohammad Memorial Hospital, Ethiopia. Science 2021;10:72–77.

[30] Mekebo GG Argawu AS Likassa HT . Factors influencing exclusive breastfeeding practice among under-six months infants in Ethiopia. BMC Pregnancy Childbirth 2022;22:1–0. 35941576 10.1186/s12884-022-04955-x PMC9361573

[31] Khadka KB Lieberman LS Giedraitis V . The socio-economic determinants of infant mortality in Nepal: analysis of Nepal Demographic Health Survey, 2011. BMC Pediatr 2015;15 1:1 1. 26459356 10.1186/s12887-015-0468-7 PMC4603581

[32] Baraki AG Akalu TY Wolde HF . Factors affecting infant mortality in the general population: evidence from the 2016 Ethiopian demographic and health survey (EDHS); a multilevel analysis. BMC Pregnancy Childbirth 2020;20:1–8. 10.1186/s12884-020-03002-x PMC7229626 32414348

[33] Tesema GA Teshale AB . Residential inequality and spatial patterns of infant mortality in Ethiopia: evidence from Ethiopian Demographic and Health Surveys. Trop Med Health 2021;49:1–5. 33499956 10.1186/s41182-021-00299-y PMC7839209

[34] Kitila FL Petros RM Jima GH . Under-five mortality and associated factors in southeastern Ethiopia. PLoS One 2021;16:e0257045. 34492085 10.1371/journal.pone.0257045 PMC8423275

[35] Roro EM Sisay MM Sibley LM . Determinants of perinatal mortality among cohorts of pregnant women in three districts of North Showa zone, Oromia Region, Ethiopia: community based nested case control study. BMC Public Health 2018;18:1 1. 10.1186/s12889-018-5757-2 PMC6052561 30021557

[36] Gobebo G Mulugeta W Yaekob T . Determinants of women unemployment: evidence from Ethiopia (Case of HalabaTown, SNNPR). Int J Dev Res 2017;7:16630–16639.

[37] Geremew BM Gelaye KA Melesse AW . Factors affecting under-five mortality in ethiopia: a multilevel negative binomial model. Pediatr Health Med Ther 2020;11:525. 10.2147/PHMT.S290715 PMC7781031 33408551

[38] Tadesse Zeleke A . Effect of health extension service on under-five child mortality and determinants of under-five child mortality in Derra district, Oromia regional state, Ethiopia: a cross-sectional study. SAGE Open Med 2022;10:20503121221100610. 35646353 10.1177/20503121221100610 PMC9134460

[39] Fenta SM Ayenew GM Fenta HM . Community and individual level determinants of infant mortality in rural Ethiopia using data from 2016 Ethiopian demographic and health survey. Sci Rep 2022;12:16879. 36207579 10.1038/s41598-022-21438-3 PMC9546827

[40] Adebe KL Wake SK Mekebo GG . Analysis of regional heterogeneity and determinants of perinatal mortality in Ethiopia. Ann Med Surg. 2023;85:902–907. 10.1097/MS9.0000000000000400 PMC10129084 37113814

[41] Kibret GD Demant D Hayen A . The effect of distance to health facility on neonatal mortality in Ethiopia. BMC Health Serv Res 2023;23:114. 36737761 10.1186/s12913-023-09070-x PMC9896723

[42] Osei-Poku GK Mwananyanda L Elliott PA . The apparent burden of unexplained sudden infant deaths in Lusaka, Zambia: findings from analysis of verbal autopsies. Gates Open Res 2023;7:46.

[43] Deressa AT Desta MS . Prevalence and determinants of neonatal near miss in Ethiopia: a systematic review and meta-analysis. PLoS One 2023;18:e0278741. 36809252 10.1371/journal.pone.0278741 PMC9942950

[44] Ahmed AT Farah AE Ali HN . Determinants of early neonatal mortality (hospital based retrospective cohort study in Somali region of Ethiopia). Scie Rep 2023;13:1114. 10.1038/s41598-023-28357-x PMC9859816 36670231

[45] Gudayu TW . Epidemiology of neonatal mortality: a spatial and multilevel analysis of the 2019 mini-Ethiopian demographic and health survey data. BMC Pediatr 2023;23:1–4. 36647037 10.1186/s12887-023-03838-0 PMC9843859

[46] ICF, E.P.H.I.a., Ethiopia Mini Demographic and Health Survey 2019: Final Report. 2021, EPHI and ICF: Rockville, Maryland, USA.

[47] Kiross GT Chojenta C Barker D . Individual-, household-and community-level determinants of infant mortality in Ethiopia. PLoS One 2021;16:e0248501. 33711062 10.1371/journal.pone.0248501 PMC7954351

[48] Katz J West KP Jr Khatry SK . Risk factors for early infant mortality in Sarlahi district, Nepal. Bull World Health Organ 2003;81:717–725. 14758431 PMC2572324

[49] Suri K Bhandari V Lerer T . Morbidity and mortality of preterm twins and higher-order multiple births. J Perinatol 2001;21:293–299. 11536022 10.1038/sj.jp.7200492

[50] Asif MF Pervaiz Z Afridi JR . Socio-economic determinants of child mortality in Pakistan and the moderating role of household’s wealth index. BMC Pediatr 2022;22:1–8. 34980031 10.1186/s12887-021-03076-2 PMC8722329

[51] Nattey C Masanja H Klipstein-Grobusch K . Relationship between household socio-economic status and under-five mortality in Rufiji DSS, Tanzania. Glob Health Action 2013;6:19278. 23364083 10.3402/gha.v6i0.19278 PMC3556682

